# Molecular Characterization of Human Rotavirus from Children with Diarrhoeal Disease in Sokoto State, Nigeria

**DOI:** 10.1155/2016/1876065

**Published:** 2016-03-09

**Authors:** B. R. Alkali, A. I. Daneji, A. A. Magaji, L. S. Bilbis, F. Bande

**Affiliations:** ^1^Department of Veterinary Microbiology, Faculty of Veterinary Medicine, Usmanu Danfodiyo University, PMB 2346, Sokoto, Nigeria; ^2^Department of Veterinary Microbiology, Usmanu Danfodiyo University, PMB 2346, Sokoto, Nigeria; ^3^Department of Biochemistry, Faculty of Science, Usmanu Danfodiyo University, Sokoto, Nigeria; ^4^Department of Veterinary Services, Ministry of Animal Health and Fisheries Development, Usman Faruk Secretariate, Sokoto, Nigeria

## Abstract

This study was conducted to detect and characterize prevalent human group A rotavirus strains from 200 diarrheic children in Sokoto, Nigeria, by ELISA, monoclonal antibody (Mab) serotyping and Reverse Transcription-Polymerase Chain Reaction (RT-PCR) techniques. Rotavirus was detected in 25.5% of the children. The G-serotypes observed in circulation were G4: 16 (59.3%), G1: 4 (14.8%), G2: 3 (11.1%), G3: 3 (11.1%), and G12: 1 (3.7%). The monoclonal antibody (Mab) serotyping detected G1 and G3 but did not detect G4 and G2 serotypes. The Mab typing of the G1 and G3 serotypes was consistent with the result of the RT-PCR. The VP4 genotypes detected were P[6] 3 (13%), P[8] 11 (47.8%), and the rare human P genotype (P[9]), found in 9 patients (39.1%). Nine strains identified with the common G and P combinations were G4 P[8] 5 (56%), G4 P[6] 1 (11%), G1 P[8] 2 (22%), and G3 P[8] 1 (11%), while seven strains with unusual combinations or rare G or P genotypes identified were G12 P[8] 1 (14%), G2 P[8] 2 (29%), and G4 P[9] 4 (57%). To our knowledge this is the first molecular study of human rotavirus and report of rare human G and P serotypes in Sokoto State.

## 1. Introduction

Rotavirus is a nonenveloped, segmented RNA virus classified under the family Reoviridae. Rotavirus constitutes a major cause of severe gastroenteritis in young children worldwide [1–3]. Studies have shown that, by the age of two years, almost all children stand the risk of being infected with rotavirus, with children living in the industrialized countries experiencing their first infection at comparatively older age compared to those in developing countries [4, 5].

In sub-Saharan Africa, gastroenteritis remains a major cause of childhood morbidity and mortality [6] and a leading cause of childhood illness as a result of poor economy, infrastructure, and political instability [7]. In a review of 43 studies from 15 countries, including Nigeria, Cunliffe et al. [4] reported that, of the 25 million children born each year in sub-Saharan Africa, 4.3 million (about 1 in 6) dies by the age of 5 years and about 1/5 of these deaths (850,000) are associated with diarrhoea. Among all the identified causes of infantile diarrhoea, rotavirus has been ranked as the single most important pathogen associated with diarrhoea in both hospitalized and outpatient cases. In Nigeria, for example, a high incidence of childhood diarrhoea is estimated to account for over 160 000 of all deaths in children less than 5 years of age annually and approximately 20% are associated with rotavirus infection [8].

Although it was speculated that an effective and properly administered rotavirus vaccine in Africa could potentially prevent 170,000–210,000 deaths (about 1 in 20) annually based on the assumptions that 20–25% of all childhood diarrhoea deaths are due to rotavirus [4], there is a lack of information on the epidemiology and genetic characteristics of the circulating human retrovirus in most of the developing nations. This will be crucial to guide control and prevention strategies so as to ensure that rotavirus infection is reduced. Thus, the objectives of this study are to determine the occurrence and molecular characteristics of human rotavirus among children in Sokoto State, Nigeria.

## 2. Material and Methods

### 2.1. Study Area

Samples were collected from major hospitals in Sokoto which lies between longitude 11° 30′ to 13° 50′ East and latitude 4° to 6′ North. The GNP per capita in the state was put at 320 dollars [9].

### 2.2. Sampling Method

A simple random sampling method was adopted in the study. The formula of Campbell [10] was used to estimate the minimum of 189 samples; however, in order to increase the precision and chances for the detection of infection, the samples were increased to 200.

### 2.3. Sample Collection

Diarrhoea samples were collected from diarrheic children less than 5 years of age presented at the study hospitals. The samples were obtained following parental consent and ethical approval from the medical research ethics committee of the hospitals. Diarrhoea in this study was defined as the passage of more than 3 looser than normal stools within 24 hours. The stool samples were collected aseptically in sterile commercial bijou bottles adequately labeled (patient ID and date of collection) and transported on ice to the Veterinary Microbiology Laboratory of Usmanu Danfodiyo University, Sokoto, where they were stored at −20°C until being transported on ice to the Noguchi Memorial Institute for Medical Research (NMIMR) Accra, Ghana, where they were also stored at −20°C until tested.

### 2.4. Preparation of 10% Stool Suspension

The stored stool specimens were retrieved after freeze thawing and a 10% stool suspension was prepared by pipetting 1.5 mL of supply specimen preparation buffer (included in the kit).

### 2.5. Detection of Rotavirus Antigen by ELISA

Rotavirus antigens in stool samples were detected by a commercially available DAKO Rotavirus ELISA kit according to the manufacturer's instructions. The result was read spectrophotometrically in 30 minutes after stopping the reaction on Multiskan ELISA reader (Multiskan Plus, Labsystems Helsinki, Finland) at the reference wavelength of 450 nm.

### 2.6. Sodium Dodecyl Sulphate Polyacrylamide Gel Electrophoresis (SDS-PAGE)

All ELISA positive samples (*n* = 51) were subjected to SDS-PAGE according to the technique described by Herring et al. [11].

### 2.7. Reverse Transcription-Polymerase Chain Reaction (RT-PCR)

The G-typing was conducted according to the method of Gouvea et al. [12] and Das et al. [13] whilst the P-typing was conducted as described by Gentsch et al. [14]. The protocols for viral RNA extraction from the stools and the RT-PCR typing protocols for rotavirus gene 7 (G-serotyping) and gene 4 (P-genotyping) were kindly provided by Dr. Gentsch of the Viral Gastroenteritis Section, CDC, Atlanta, USA.

### 2.8. Monoclonal Antibody (Mab) Serotyping

Mab serotyping was carried out with monoclonal antibodies specific for group A human rotavirus strains using the Mab serotyping kit according to Manufacturer's instructions. The Mab serotyping kit was kindly provided by the National Institute of Health, Atlanta, Georgia, USA. The method employed the ELISA protocol and the procedure was basically as described by Taniguchi et al. [15] with the monoclonal antibody as the capture reagent but skim milk was used to reduce the background as described by Coulson et al. [16].

### 2.9. VP7 and VP4 Genotyping

Phenol/chloroform method described by Steele and Alexander [17] was used to extract the viral RNA from all PAGE positive specimens and then purified with the RNaid® Kit (Bio 191, Carlsbad, USA). The extracted RNA was stored at −20°C until further use.

The VP7 and VP4 genotyping was performed as described by Gouvea et al. [12] and Gentsch et al. [14]. Briefly, viral RNA was subjected to Reverse Transcription (RT) step and Polymerase Chain Reaction (PCR) step was performed to amplify the transcribed gene. Finally a seminested PCR was performed using genotype specific primers as a mean to determine the viral genotype (Table 1).

### 2.10. VP7 First Round Amplification

The purified viral RNA was reverse transcribed according to the method of Gouvea et al. [12]. Briefly, 1 *μ*L each of the specific primer pair (sBeg9/End9) was added to 8 *μ*L of previously extracted dsRNA and put in a 500 *μ*L PCR tube. The solution was heated for 5 minutes at 94°C to denature the dsRNA and chilled immediately in an ice bath for 2 minutes. The denatured dsRNAs were then reverse transcribed by adding 3.2 *μ*L of master mix (0.2 *μ*L of 10 mM dATP, 0.2 *μ*L of 10 mM dCTP, 0.2 *μ*L of 10 mM dGTP, 0.2 *μ*L of 10 mM dTTP, 0.4 *μ*L of avian myeloblastosis (AMV), reverse transcriptase, and 20 *μ*L of 5x AMV buffer) and incubated for 20 minutes at 42°C in a water bath. The cDNA was then amplified by PCR in a 40 *μ*L reaction mixture containing 1 *μ*L of 10 mM dATP, 1 *μ*L of 10 mM dCTP, 1 *μ*L of 10 mM dGTP, 1 *μ*L of 10 mM dTTP, 4 *μ*L of 10x Taq buffer, 2.4 *μ*L of 25 mM MgC1_2_, 30 *μ*L of double distilled water, and 0.3 *μ*L Taq polymerase prior to use. No template control (NTC) included negative control. Thirty cycles of PCR (1 minute denaturing at 94°C, 2 minutes annealing at 42°C, and 3 minutes extension at 72°C) and a final extension cycle at 72°C for 7 minutes was performed on a Gene AMP PCR Primus 25 System machine The amplified samples were analysed onto 2% agarose (SEAKEM, USA) and visualised on the GelDOC system.

### 2.11. VP7 Second Round Amplification

A second round Polymerase Chain Reaction was carried out to determine the genotype of the rotavirus strains. Briefly, 1 *μ*L of the first round reaction products was added to 40 *μ*L PCR-MM containing 1 *μ*L of each of six serotype-specific primers (aBT-1, aCT-2, aET-3, aDT-4, aAT-8, and aFT-9) and the primer RVG9, 1 *μ*L of 10 mM dATP, 1 *μ*L of 10 mM dCTP, 1 *μ*L of 10 mM dGTP, 1 *μ*L of 10 mM dTTP, 4 *μ*L of 10x Taq buffer, 2.4 *μ*L of 25 mM MgCl_2_, 30 *μ*L of ddH_2_O, and 0.3 *μ*L of Taq polymerase and PCR performed according to the condition stated above. The amplified products were analysed on 2% agarose gel and the genotype determined based on the size of the resultant amplicon.

### 2.12. VP4 (VP8^*∗*^) Genotyping

**Step 1 .**

RT-PCR of the VP4 gene used terminal primers Con2 and Con3.

**Step 2 .**

Genotyping of VP4 gene of human rotaviruses used a cocktail of Gentsch primers, Con3 + [1T-1, 2T-1, 3T-1, 4T-1, and 5T-1], to determine genotypes P[8], P[4], P[6], P[9], and P[10], respectively (Table 2). Amplification for both sets of primers was carried out by initial denaturation at 94°C for 2 mins, denaturation at 94°C for 1 minute, annealing at 42°C for 2 minutes, extension at 72°C for 5 minutes, and final extension at 72°C for 7 minutes. 30 cycles of repetitions were carried out.

### 2.13. VP4 First Round Amplification

The RT-PCR typing method used for P-typing was similar to that used for G-typing. However, different primer pairs were used instead. For P-typing, the consensus primers used were Con2 and Con3 as described by Gentsch et al. [14]. The PRC conditions were also as described for G-typing.

### 2.14. VP4 Second Round Amplification

A second round Polymerase Chain Reaction to determine the P-types of the rotavirus strains was similarly performed using 5 P genotype-specific primers (1-T1, 2T-1, 3T-1, 4T-1, 5T-1) and the primer Con3. The PCR amplification was then performed according to the conditions earlier stated for G-typing. The amplified products were analysed on 2% agarose gel and the genotype determined from the size of the amplicons.

### 2.15. Interpretation of RT-PCR Genotyping for Human Rotavirus

Table 3 provides the expected band sizes of different G- and P-types used as a criteria for interpretation of human rotavirus G- and P-types results based on the primer types used (Table 3).

## 3. Result

### 3.1. Rate of Rotavirus Detection among Children in Sokoto, Nigeria

Out of the 200 human diarrhoea stools examined by ELISA, rotavirus was detected in 51 of the samples, thus indicating a prevalence of 25.5%.

### 3.2. Polyacrylamide Gel Electrophoresis (PAGE)

**Figure 1:**
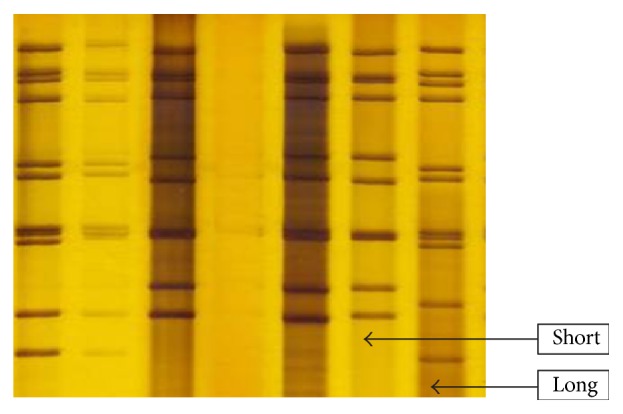
Representative electrophoretic patterns of human stool specimens analysed by PAGE with their short and long electropherotypes.

Of the 51 human ELISA positive samples tested by PAGE, 38 (74.5%) yielded electrophoretic patterns typical of rotavirus. 29 of the rotavirus strains (76.3%) had classical long rotavirus RNA electrophoretic patterns and 9 (23.7%) exhibited classical short RNA profile as shown in (Table 4). The representative of human electrophoretic patterns of rotavirus strains from rotavirus strains from the SDS-PAGE results is shown in Figure 1.

### 3.3. Rotavirus VP7 Genotype Analysis

#### 3.3.1. Rotavirus VP7 and VP4 Genotype Analysis

**Figure 2:**
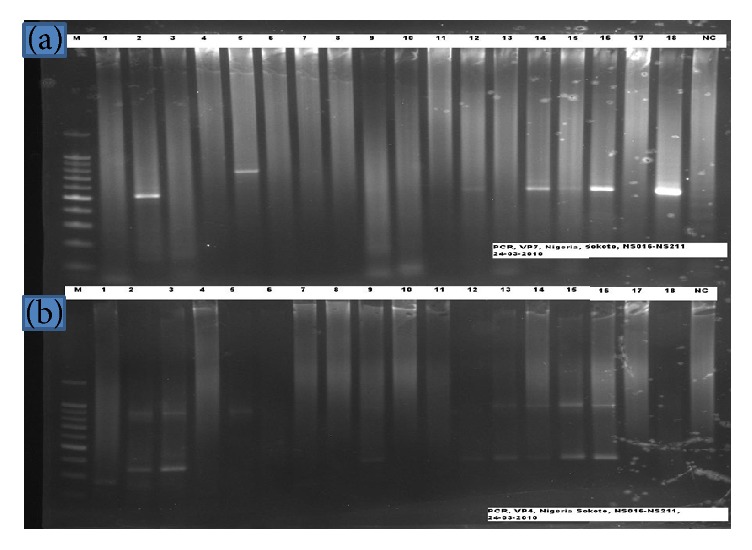
Illustration of VP7 and VP4 genotype on agarose gel. Lane 1 (from left to right) shows 1 kb Plus DNA Marker. The Gel Picture was divided into two. (a) was for VP7 samples and (b) was for VP4 samples (PCR). Lane l labeled “M” is for the molecular marker or Ladder. The last lane labeled “NC” contained the negative control.

Of the 38 PAGE positive samples further subjected to PCR genotyping method, first round RT-PCR revealed a total of twenty-nine (76.3%) positives for VP7 genotype. Following second round amplification, 27 (71.1%) of the rotavirus isolates were successfully assigned a VP7 (G-type) specificity (Figure 2(a)), while 11 (28.9%) of the isolates were nontypeable. Similarly, VP4 genotyping of the 38 PAGE positive samples after first round amplification showed 27 (71.1%) positives for VP4 genotype. However, only 23 (60.5%) samples produced second round PCR products that could be assigned a VP4 genotype (Figure 2(b)).

### 3.4. Predominant V7-Genotypes Circulating in Sokoto

**Figure 3:**
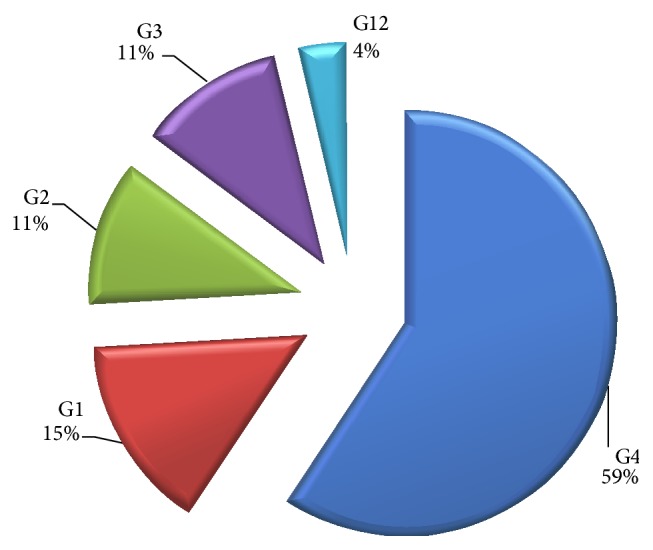
Predominant G genotypes circulating in Sokoto metropolis.

Five different rotavirus VP7 genotypes including the rare human rotavirus G genotype (G12) were detected. The five predominant genotypes observed in circulation were sixteen G4 (59.3%), four G1 (14.8%), three G2 (11.1%), three G3 (11.1%), and one G12 (3.7%) (Figure 3). G1, G2, and G3 are both common human G-types while the G12 is a rare human G-type. The rare G-serotype could be animal rotavirus strains that accidently infected human or reassortant viruses between animal and human rotavirus generated in nature.

### 3.5. Analysis of Electrophoretic Profiles of VP7 Genotypes

The distribution of VP7 serotypes according to electrophoretic profile of the isolates is summarized in Table 5. Twenty-nine samples exhibited classical long RNA electrophoretic profile on PAGE and 22 (75.9%) were typed and assigned to particular G-types while 7 (24.1%) could not be assigned any VP7 genotype. Thirteen (59.1%) of these were typed as G4, three (13.6%) as G1, three (13.6%) as G2, two (9.1%) as G3, and one (4.5%) as G12. Nine samples exhibited classical short RNA electrophoretic profile on PAGE and 5 (55.6%) were typed and assigned to particular G-types while 4 (44.4%) could not be assigned any VP7 genotype. Of the 5 that were typed, three (60%) were typed as G4, one as G3 (20%), and one (20%) as G1.

### 3.6. Monoclonal Antibody Serotyping

The monoclonal antibody serotyping detected G1 and G3 with consistency both on visual examination and spectrophotometrically. The OD values of the assigned serotypes were twice greater than the values obtained from the samples with the same Mab. The Mab typing of the G1 and G3 serotypes was consistent with the result of the RT-PCR serotyping of these types.

### 3.7. VP4 Genotype Analysis

**Figure 4:**
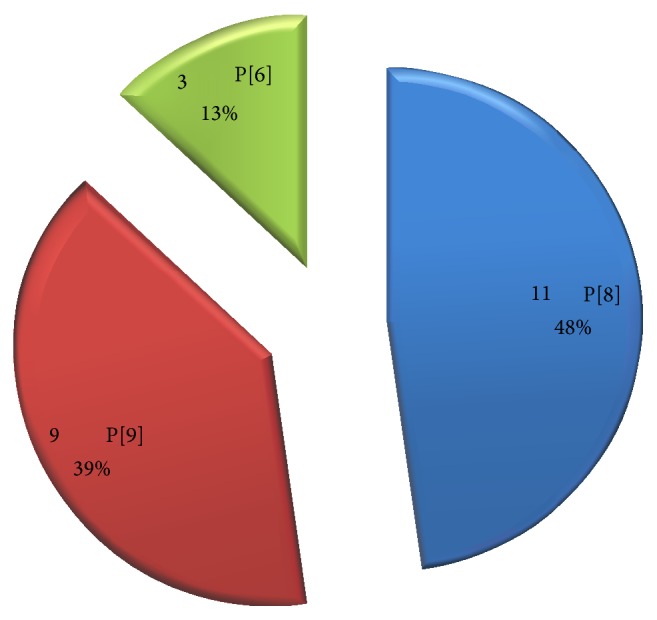
Predominant P genotypes circulating in Sokoto, Nigeria.

The VP4 genotypes detected during the study included two of the recognized human rotavirus VP4 gene alleles, P[6] and P[8], and a rare human P[9]. The most predominant P4 genotype was P[8] 11 (47.8%), followed by P[9] 9 (39.1%) and P[6] 3 (13%), and none of the strains was carrying a P[4] VP4 gene (Figure 4). The data showed a high percentage of rare P genotype, P[9] which is considered as an indication of either animal rotavirus strains that accidently infected human or reassortant viruses between animal and human rotavirus generated in nature.

### 3.8. Occurrence of G and P Genotypes Combinations among Children in Sokoto

**Figure 5:**
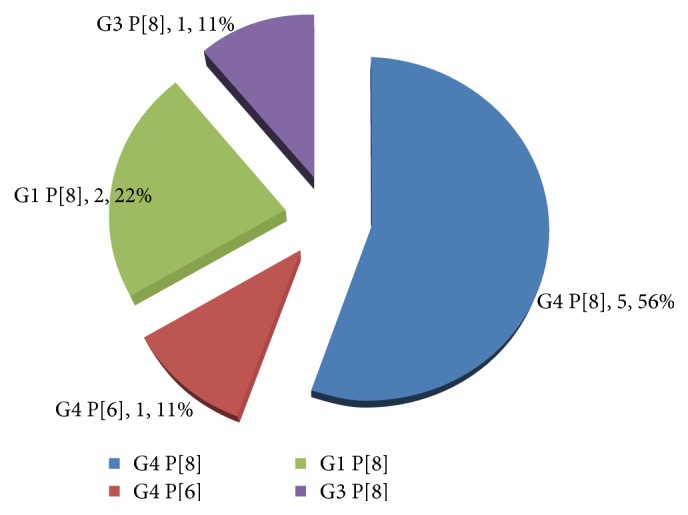
Four common G/P-types combinations circulating in Sokoto metropolis.

Four distinct strains were identified with the common G and P combinations being strains bearing the genotype G4 P[8] (56%), G4 P[6] (11%), G1 P[8] (22%), and G3 P[8] (11%) (Figure 5).

**Figure 6:**
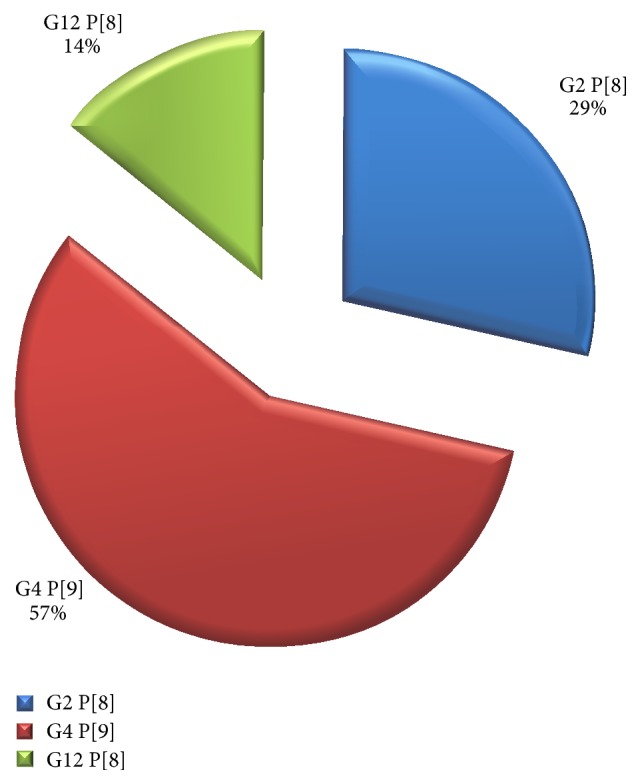
Three uncommon G/P-types combinations circulating in Sokoto metropolis.

Similarly, three distinct strains with unusual combinations or rare G or P genotypes were observed; these included G12 P[8] (14%), G2 P[8] (29%), and G4 P[9] (57%) as summarized in Figure 6.

## 4. Discussions

Rotavirus has been identified to be the single most important pathogen associated with diarrhoea cases in both hospital patients and outpatients [4, 18]. In this study, 51 (25.5%) out of the 200 diarrhoeic children tested were found to be positive for rotavirus while 149/200 (74.5%) tested negative for rotavirus. Thus, the prevalence of rotavirus diarrhea accounted for 25.5% of diarrhea cases among children younger than five years presented to hospitals in Sokoto metropolis. The result of this study is consistent with the sentinel based rotavirus surveillance system and hospital based study results within the African region [19].

Interestingly, earlier studies carried out in different parts of the northern Nigeria reported low prevalence. The report of Pennap and Umoh [20] showed rotavirus infection prevalence of 15.6% among children 0–60 months old that were presented with diarrhea in northeastern Nigeria. Aminu et al. [21] similarly reported rotavirus prevalence of 18% among diarrheic children and 7.2% among nondiarrheic children in a hospital setting in northern Nigeria and prevalence of 9% in children under five years of age in a community based study in the same region. Similarly, other investigators reported lower prevalence of the infection in the northern region [22]. The low prevalence reported in the community based study is expected as higher prevalence of rotavirus infection more likely to be encountered in hospital based studies since rotavirus positive cases are often severe and likely represented in hospitals [23]. However, generally, studies from southern Nigeria had shown higher rotavirus prevalence values than those from northern Nigeria [24–27]. The differences in the prevalence recorded by different investigators had been attributed to differences in time of sample collection, method of screening samples, geographical location of the study, or changing trends of the burden of the rotavirus disease over the years [28].

The possibility of direct transmission of animal rotavirus to human host and the uncommon serotypes detected in this study may explain the high prevalence of the disease among children with often contact with animals.

The analysis of electropherotypes of rotavirus isolates provides information on genetic diversity of the virus and heterogeneity of circulating strains and can be useful in tracing spread through a population. Indeed, viruses of the same serotype could exhibit different electropherotypes and those of the same electropherotypes different serotypes [29]. In this study, analyses using PAGE showed that, of the 51 human ELISA positive samples tested by PAGE, 38 (74.5%) yielded electrophoretic patterns typical of rotavirus while 13 showed no profile. Of the 38 rotavirus strains that yielded RNA profile, 29 (76.3%) had classical long rotavirus RNA electrophoretic patterns and 9 (23.7%) exhibited classical short RNA profile. The inconsistency between ELISA results and PAGE results might be as a result of RNA degradation in which case strains will not yield RNA profile on PAGE. The absence of RNA bands had also been attributed to too little RNA or its degradation during the phenol/chloroform extraction stage [30]. Strains with long RNA profiles were the most prevalent strains in circulation in the study area. This is in consonant with the study of Aminu et al. [21]. Interestingly, however, there were no unusual electropherotypes even in the samples containing the G12 strains. This observation was similar to that of Adah et al. [22].

The RT-PCR VP7 serotyping results revealed that twenty-seven (71.1%) of the rotavirus isolates were successfully assigned a VP7 (G-type) specificity. While 11 (28.9%) of the isolates were nontypeable. It was likely that the nontypeable strains did not contain enough RNA to permit typing similar to the observation of Adah et al. [22]. It may also be as a result of the existence of serotypes which the serotype-specific primers used in the study could not detect.

The monoclonal antibody serotyping detected G1 and G3 with consistency both on visual examination and spectrophotometrically. But no other G-serotype was typed using Mab apart from the two serotypes (G1 and G3). The OD value recorded against each of these two serotypes with the specific Mab was consistently more than twice than what was recorded in the reaction of other samples with the same Mab. The Mab typing of the G1 and G3 serotypes was consistent with the result of the RT-PCR serotyping of these types.

Previous studies had shown that the most common global human G-serotypes were G1, G2, G3, G4, and G9, with G1 being most prevalent and G9 the fastest emerging genotype worldwide [31–33]. In this study, five different rotavirus VP7 serotypes including the rare human rotavirus G-serotype (G12) were detected. The predominant serotypes observed in circulation in this study were sixteen G4 (59.3%), four G1 (14.8%), three G2 (11.1%), three G3 (11.1%), and one G12 (3.7%). In earlier studies reported by Adah et al. [22] and Pennap et al. [30], no G2 or G4 serotypes were detected; however, Aminu et al. [21] had recently reported the detection of G2 serotypes in northern Nigeria. The absence of G9 serotype in this study could be a result of genetic shift since G9 serotype was previously reported in Northern region of the country [21, 22].

Interestingly, we detected a rare G12 serotype detected in a single infection. This is consistent with the studies reported in Thailand [34] and India [35]. Although G12 rotaviruses were first identified in the Philippines in 1987, it emerged over the past few years in numerous countries [36] with the first incidence in Africa reported in Johannesburg, South Africa [37]. Indeed, the detection of G12 serotype may suggest either accidental human infection by animal rotavirus strains or reassortant viruses between animal and human rotavirus generated in nature. Already rotavirus gene reassortment between human and bovine strains has been reported previously [22].

The results of VP4 genotyping revealed that the VP4 genotypes detected during the study included two of the recognized human rotavirus VP4 gene alleles, P[6] and P[8], and a rare human P genotype (P[9]). The most predominant P-type detected in this study was P[8] 11 (47.8%) followed by P[9] 9 (39.1%) and P[6] 3 (13%). The predominance of P[8] strains in this study agreed with the worldwide distribution of this genotype as epidemiological studies had shown P[8] strains to be the most commonly identified worldwide [38, 39]. It is noted that similar to the result of the study conducted in Nigeria by of Adah et al. [22], none of the strains carrying P[4] VP4 gene was found. However, in disagreement with the work of Adah et al. [22], in which P[6] was reported as the predominant type and P[8] as the second most common type, P[6] strains were the least detected in this study and P[8] was the most predominant. In fact, P[6] genotype was previously thought to be associated with neonatal rotavirus strains and hence thought to be associated with asymptomatic infection [40]. But in this study, it was associated with symptomatic diarrhea similar to what was observed in earlier studies conducted in Cameroon [41], Nigeria [21, 22], and Ghana [42]. This implies that this genotype is now becoming more associated with symptomatic diarrhea. In fact, rotavirus with VP4 P[6] genotype was the only genotype detected in the study conducted by Aminu et al. [21].

The prevalence of human rotaviruses with P[9] had been shown to be low. For example, in the surveys which detected P[9] strains, only 0.2% of 1,316 rotaviruses between 1996 and 1999 in the United States [43] and 3.8% of 282 rotaviruses between 1991 and 1994 in Israel were of this P[9] specificity. Furthermore, all the strains with P[9] specificity had been associated with G3 or G1 specificity [44, 45]. Therefore, the high percentage of this genotype (P[9]) detected in this study is considered as an indication of either animal rotavirus strains that accidently infected human or reassortant viruses between animal and human rotavirus generated in nature.

Studies in many countries have shown that G1 P[8], G2 P[4], G3 P[8], and G2 P[6] are the G-P combinations most commonly found worldwide [38, 39, 46]. In this study, nine distinct strains were identified with the common G and P combinations bearing the combination G4 P[8] (56%), G4 P[6] (11%), G1 P[8] (22%), and G3 P[8] (11%). Similarly, seven distinct strains with unusual combinations or rare G or P genotypes were observed. These were G12 P[8] (14%), G2 P[8] (29%), and G4 P[9] (57%).

Numerous studies had reported an unexpectedly high diversity of rotavirus strains in most developing and developed countries including Nigeria [34, 39, 42, 47–54].

This had been attributed to natural reassortment which appears to be detected more frequently in developing countries than in developed world owing to low levels of hygiene and poor immunological defence in infants which facilitate mixed infections and hence more reassortment. In addition more close contact among humans, livestock, and other animals in developing countries makes the possibility of emergence of virulent rotavirus strains very high as a result of gene reassortment [55]. Apart from gene reassortment, interspecies transmissions of rotaviruses involving a whole genome constellation evidenced by molecular characterization of rotaviruses isolated from different species have been suggested [3].

Indeed, studies had indicated that uncommon human rotavirus strains are emerging as global strains, which has important implications for effective vaccine development [4, 56]. The detection of unusual G/P combinations in the present study adds to this pool of information and further confirms the emergence of these unusual strains.

## 5. Conclusions

The study reported a high prevalence of rotavirus infection among diarrheic children attending health care in Sokoto State, Nigeria. Both common, uncommon, and combinations of various genotypes were identified as revealed by their electrophoresis pattern based on the VP4 and VP7 gene. Overall, this study contributed to the global understanding of the molecular epidemiology of human rotavirus which will be useful in guiding the choice and application of rotavirus vaccines for effective control and preventions.

## Figures and Tables

**Table 1:** List of VP7 primers used for VP7 (G) genotyping.

Primer designation	Sequences (5′-3′)	Region amplified	Amplicon size
Beg9	GGCTTTAAAAGAGAGAATTTCCGTCTGG	1–29	
End9	GGTACACATCATACAATTCTAATCTAAG	1062–1036	
RVG9	GGTACATCATACAATTCT	1062–1044	
aAT8-69M (G8)	GTCACACCATTTGTAAATTCG	178–198	885 bp
aBT1-Wa (G1)	CAAGTACTCAAATCAATGATGG	314–335	749 bp
aCT2-DS-1 (G2)	CAATGATATTAACACATTTTCTGTG	411–435	652 bp
aDT4-ST-3 (G4)	CGTTTCTGGTGAGGAGTTG	480–498	583 bp
aET3-P (G3)	CGTTTGAAGAAGTTGCAACAG	689–709	374 bp
aFT9-W161 (G9)	CTAGATGTAACTACAACTAC	757–776	306 bp
G10-(G10)	ATGTCAGACTACARATACTGG	666–687	397 bp
G12-(G12)	CCGATGGACGTAACGTTGTA	548–567	515 bp

Source: Noguchi Memorial Institute for Medical Research (NMIMR).

**Table 2:** Primers used for VP4 genotyping of human rotavirus.

Primer designation	Sequence (5′-3′)	Nucleotide position	Genotype
Con2	ATTTCGGACCATTTATAACC	868–887	—
Con3	TGGCTTCGCTCATTTATAGACA	11–32	—
1-TI-KU	ACTTGGATAACGTGC	336–356	P[8]
2T-1	CTATTGTTAGAGGTTAGAGTC	474–494	P[4]
3T-1	TGTTGATTAGTTGGATTCAA	259–278	P[6]
4T-1	TGAGACATGCAATTGGAC	385–402	P[9]
5T-1	ATCATAGTTAGTAGTCGG	575–594	P[10]

Source: Noguchi Memorial Institute for Medical Research (NMIMR).

**Table 3:** Primers and expected band sizes for G/P-types on gel primers.

Genotype (G-types)	Amplicon size (bp)	Gentsch Cocktail + Con3 [P-type]	Size (bp)
G1	749	P[4]	483
G2	625	P[6]	267
G3	374	P[8]	345
G4	583	P[9]	391
G8	885	P[10]	594
G9	306		
G10	397		
G12	512		
Beg9/End9	1062		

Source: Noguchi Memorial Institute for Medical Research (NMIMR).

**Table 4:** PAGE distribution of electropherotypes of human rotavirus strains in Sokoto.

Electropherotypes	Total	Percentage (%)
Long	29	76.3
Short	9	23.7

Total	38	100

**Table 5:** Distribution of VP7 serotypes according to electrophoretic profile.

VP7 serotype	Number of VP7 serotypes	Long electropherotype	Short electropherotype	% Long electropherotype	% Short electropherotype
G1	4	3	1	13.6	20
G2	3	3	0	13.6	0
G3	3	2	1	9.1	20
G4	16	13	3	59.1	60
G12	1	1	0	4.5	0

Total	27	22	5	99.9	100
